# One-step production of C6–C8 carboxylates by mixed culture solely grown on CO

**DOI:** 10.1186/s13068-017-1005-8

**Published:** 2018-01-09

**Authors:** Pinjing He, Wenhao Han, Liming Shao, Fan Lü

**Affiliations:** 1 State Key Laboratory of Pollution Control and Resource Reuse, Tongji University, Shanghai, 200092 People’s Republic of China; 2 Institute of Waste Treatment and Reclamation, Tongji University, Shanghai, 200092 People’s Republic of China; 3 Centre for the Technology Research and Training on Household Waste in Small Towns & Rural Area, Ministry of Housing and Urban–Rural Development of P. R. China (MOHURD), Shanghai, 200092 People’s Republic of China; 4 Shanghai Institute of Pollution Control and Ecological Security, Shanghai, 200092 People’s Republic of China

**Keywords:** Syngas fermentation, Chain elongation, Carboxylates, One-step production, Mixed culture

## Abstract

**Background:**

This study aimed at producing C6–C8 medium-chain carboxylates (MCCAs) directly from gaseous CO using mixed culture. The yield and C2–C8 product composition were investigated when CO was continuously fed with gradually increasing partial pressure.

**Results:**

The maximal concentrations of *n*-caproate, *n*-heptylate, and *n*-caprylate were 1.892, 1.635, and 1.033 mmol L^−1^, which were achieved at the maximal production rates of 0.276, 0.442, and 0.112 mmol L^−1^ day^−1^, respectively. Microbial analysis revealed that long-term acclimation and high CO partial pressure were important to establish a CO-tolerant and CO-utilizing chain-elongating microbiome, rich in *Acinetobacter*, *Alcaligenes,* and Rhodobacteraceae and capable of forming MCCAs solely from CO.

**Conclusions:**

These results demonstrated that carboxylate and syngas platform could be integrated in a shared growth vessel, and could be a promising one-step technique to convert gaseous syngas to preferable liquid biochemicals, thereby avoiding the necessity to coordinate syngas fermentation to short-chain carboxylates and short-to-medium-chain elongation. Thus, this method could provide an alternative solution for the utilization of waste-derived syngas and expand the resource of promising biofuels.

**Electronic supplementary material:**

The online version of this article (10.1186/s13068-017-1005-8) contains supplementary material, which is available to authorized users.

## Background

In recent times, conversion of waste to biochemicals has been a highlighted topic in the context of biorefinery industry. Products of anaerobic fermentation from waste, i.e. short-chain carboxylic acids (SCCAs) and alcohols, can be converted to more valuable medium-chain carboxylic acids (MCCAs), such as *n*-caproate and *n*-caprylate, by mixed culture (also called “open culture” or “reactor microbiomes”) under anaerobic conditions, and this reaction is known as chain elongation [1, 2]. Acetate and ethanol are the dominant products derived from syngas anaerobic fermentation, while syngas, a mixture of CO, H_2_, and CO_2_, is sustainably produced from the thermal treatment of biowaste or artificial polymers [3–5]. Therefore, it is reasonable to integrate the two bioprocesses (i.e. chain elongation and syngas fermentation) to achieve the conversion of gaseous syngas to preferable liquid biochemicals, which could provide an alternative solution for the utilization of waste-derived syngas and expand the resource of promising biofuels. Thermal treatment serves as a depolymerization pretreatment of complex waste to produce syngas (carbon source and electron donor) for anaerobic conversion into clean and easily separable biochemicals.

In a previous study, Kucek et al. [6] successfully achieved MCCAs production by mixed culture from a synthetic substrate of ethanol and acetate, which mimicked syngas fermentation products. Similarly, Vasudevan et al. [7] obtained *n*-caproic acid using mixed culture from real syngas fermentation effluent. Furthermore, Gildemyn et al. [8] converted real syngas fermentation effluent into MCCAs in continuous fermentation using pure culture of *Clostridium kluyveri*, which is one of the known model organisms for chain elongation.

Nevertheless, direct production of liquid MCCAs from syngas or the dominant component—gaseous CO is much preferable, because the process requires only one bioreactor for control and optimization. To date, only some studies have achieved this one-step production process with the combination of two pure cultures on a bench scale. For example, Diender et al. [9] accomplished MCCAs production from CO using co-culture of *Clostridium autoethanogenum* and *C. kluyveri*, which are the model organisms for syngas metabolism and chain elongation, respectively. Richter et al. [10] used co-culture of carboxydotrophic *Clostridium ljungdahlii* and chain-elongating *C. kluyveri* to produce MCCAs and corresponding alcohols. Perez et al. [11] converted carboxylic acids to their corresponding alcohols using syngas as electron donor by carboxydotrophic bacteria *C. ljungdahlii* and *C. ragsdalei*. However, pure cultures are inclined to be sensitive to environmental disturbance and susceptible to contamination, and hence, are not perfect for industrial application with regard to waste valorization [12, 13]. Esquivel-Elizondo et al. [14]. gained MCCAs production from combination of ethanol and CO, where CO was used to inhibit the methanogenesis and was regarded to contribute as potential electron donor at partial pressure between 0.11 and 0.3 atm. Zhang et al. [15] got MCCAs production from mixture of CO_2_ and H_2_ by mixed culture, which also belongs to syngas components. However, compared to easily bio-converted CO_2_ and H_2_, CO is the most toxic and becomes the limitation for the utilization of syngas by mixed culture.

Therefore, the present study aimed at producing MCCAs using mixed culture using solely gaseous CO both as carbon source and electron donor. One-step production utilizing CO at gradient partial pressures was continuously run long term for 199 days. Subsequently, the production performance and microbial community composition corresponding to the combined operation of CO fermentation and chain elongation were investigated.

## Methods

### Reactor setup and cultivation

**Fig. 1:**
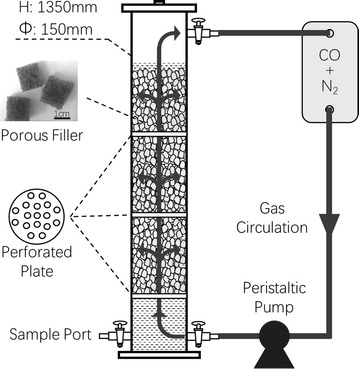
Schematic of the reactor

The reactor (Fig. 1) was made of polymethyl methacrylate, had a total volume of 21 L and effective volume of 18 L, and was purged with high-purity nitrogen gas (99.999%) to guarantee anaerobic condition. The reactor was filled with non-biodegradable, erosion-resistant sponge scouring pad, which was made of polyester fiber. The size of the fillers was about 1 cm × 1 cm × 0.5 cm, with average porosity of 96.8 ± 0.2%, average aperture of 20.12 μm, and specific surface area of 0.2063 m^2^ g^−1^. The inoculum was derived from anaerobic granular sludge of a mesophilic biogas reactor treating paper mill wastewater. The granular sludge was crushed and sieved through a 500-μm screen. The inoculum of 20 g L^−1^ volatile solids (VS) was added.

The reactor was operated in a semi-continuous mode, with continuous supply of gaseous CO and retention of liquid products. The CO served as the single carbon source and electron donor, and the partial pressure of CO in the 7-L gas bag containing the mixture of CO and N_2_ was gradually increased considering the toxicity of the gas to the mixed culture. Periodic gas circulation in upflow mode at a frequency of 15 min h^−1^ and flow rate of 300 mL min^−1^ was applied through a peristaltic pump to enhance mass transfer from gas to liquid. The gas bag was emptied and then refilled with CO–N_2_ mixture every 2 days. The basic culture medium was the same as that reported in previous studies [16, 17], except for the addition of 0.5 g L^−1^ yeast extract in the present study. The pH of the medium was only initially adjusted to 7.12 by adding 4 M HCl, and was not controlled afterwards. The mixed culture was incubated under mesophilic condition at constant temperature (35 °C) and 50 mmol L^−1^ 2-bromoethanesulfonate (2-BES) was added to it to inhibit methanogenesis [18].

Based on the gradually increased partial pressure of the supplied CO, the experiment (run for 199 days) was divided into seven phases: I (0–12 days, CO = ~0.15 atm), II (13–22 days, CO = ~0.25 atm), III (23–34 days, CO = ~0.35 atm), IV (35–46 days, CO = ~0.40 atm), V (47–60 days, CO = ~0.50 atm), VI (61–100 days, CO = ~0.60 atm), and VII (101–199 days, CO = ~0.60 atm). Phase VII was set when the MCCAs production occurred from about 100th day.

### Physiochemical analysis

Every 2 days before gas renewal, the components and volume of the gas in the gas bag were tested. The components of the gas (O_2_, H_2_, CH_4_, CO, CO_2_) were measured using a gas chromatograph (Trace1300, Thermo Fisher Scientific, Waltham, MA, USA) equipped with a flame ionization detector and thermal conductivity detector. The volume of the gas was determined by a gas meter (TG05/6, Ritter, Bochum, Germany).

Liquid samples were taken every 2 days and their pH and oxidation–reduction potential (ORP) were determined using a pH meter (PXSJ-216F, Shanghai Precision and Scientific Instrument Co., LTD, Shanghai, China) and an automatic potentiometric titrator (ZD-2, Shanghai Precision and Scientific Instrument Co., LTD, Shanghai, China), respectively. Subsequently, the samples were centrifuged at 4460*g* for 10 min in a high-speed refrigerated centrifuge (TL-18 M, Shanghai Centrifugal Machinery Research Institute, Shanghai, China), and the supernatant was diluted and acidified with 3% (v/v) phosphoric acid for carboxylates (C2–C8) analysis and just diluted for DOC, DIC and DN analysis. The concentration of carboxylates (C2–C8) was defined as the total amount of undissociated and dissociated forms of carboxylates, and was measured using a gas chromatograph (Focus GC, Thermo Scientific Co., Waltham, MA, USA) equipped with a flame ionization detector and 30 m × 0.25 mm DB-WAX UI polyethylene glycol capillary column. The dissolved organic carbon (DOC), dissolved inorganic carbon (DIC), and dissolved nitrogen (DN) contents were determined using a total carbon/total nitrogen analyzer (TOC-V_CPN_, TNM-1, Shimadzu, Kyoto, Japan). The CO consumption in moles was calculated by Clapeyron–Clausius equation: *PV* = *nRT* (*T* = 308 K). The carbon conversion efficiencies were calculated as weighted averages based on the input carbon (gaseous CO) and the carbon content of the products (mmol L^−1^).

### Microbial analysis

For microbial analysis, 10 samples were periodically collected on days 0, 10 (phase I), 20 (phase II), 34 (phase III), 44 (phase IV), 56 (phase V), 74 (phase VI), 100 (phase VI), 130 (phase VII), and 155 (phase VII), and stored at − 80 °C. The total DNA in each sample was extracted using a PowerSoil^®^ DNA isolation kit (Mo-Bio Laboratories Inc., Carlsbad, CA, USA) following manufacturer’s instructions. The quality of the DNA was assessed using gel electrophoresis (1% agarose), and the DNA concentrations were determined by NanoDrop 2000 spectrophotometer (Thermo Scientific Co., Waltham, MA, USA) (see Additional file 1: Table S1).

The variable regions V4–V5 of the microbial 16S ribosomal RNA gene were amplified by PCR using the primers ArBa515F (5′–GTGCCAGCMGCCGCGGTAA–3′) and Arch806R (5′–GGACTACHVGGGTWTCTAAT–3′), which were selected as the sequencing primers set to simultaneously obtain bacterial and archaeal information. The high-throughput sequencing was performed using Illumina HiSeq2500 platform by Majorbio Bio-pharm Technology Co., Ltd, Shanghai, China. The pretreatment and sequencing procedure were performed as described by Amato et al. [19]. The bioinformatic analyses were conducted on the online i-sanger sever (http://www.i-sanger.com/) of Majorbio Bio-pharm Technology Co., Ltd. Principle component analysis (PCA) based on Bray–Curtis distance for total microbiomes, bacteria, and archaea were conducted by the software PAST (V. 3.1.0).

## Results and discussion

### Reactor performance

**Fig. 2:**
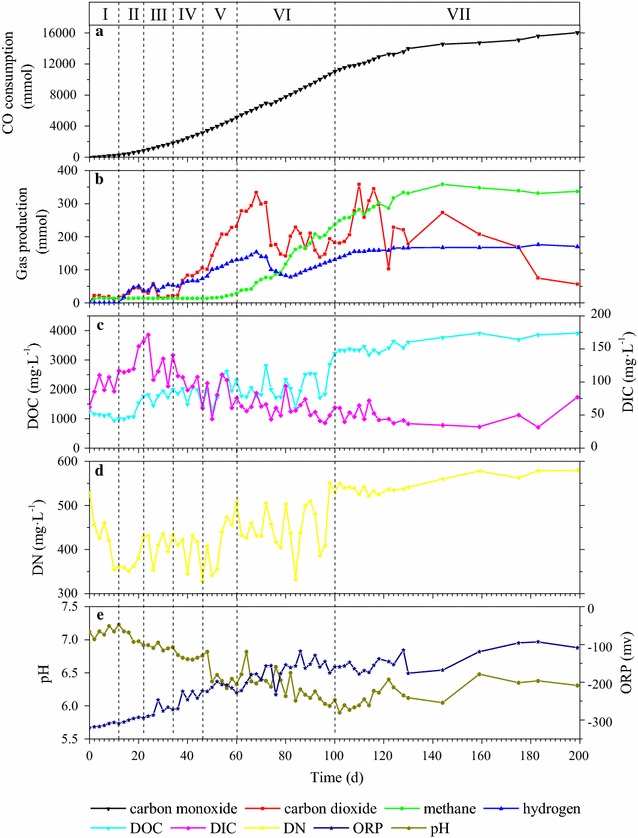
Reactor performance during the continuous operation of 199 days. **a** Cumulative CO consumption in mmol. **b** Cumulative gas production of CO_2_, H_2_, and CH_4_ in mmol. **c** DOC (dissolved organic carbon) and DIC (dissolved inorganic carbon). **d** DN (dissolved nitrogen). **e** pH and ORP (oxidation–reduction potential). The blocks labeled with I, II, III, IV, V, VI and VII indicate the seven phases during continuous feeding

**Fig. 3:**
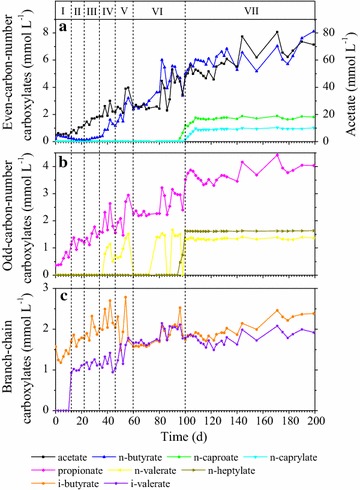
Concentration of carboxylate products in the reactor. **a** Even-carbon-number carboxylates (acetate, *n*-butyrate, *n*-caproate and *n*-caprylate). **b** Odd-carbon-number carboxylates (propionate, *n*-valerate and *n*-heptylate); **c** Branch-chain carboxylates (*i*-butyrate and *i*-valerate). The blocks labeled with I, II, III, IV, V, VI and VII indicate the seven phases during continuous feeding

The reactor performance indicated by cumulative CO consumption, cumulative biogas production, DOC, DIC, DN, pH, and ORP is shown in Fig. 2. During all the seven phases, a total of 16,025 mmol CO was consumed, while only 170 mmol H_2_, 56 mmol CO_2_, and 337 mmol CH_4_ were produced as biogas (Fig. 2b). Therefore, the carbon loss during conversion into biogas was negligible and only contributed to 2.4%. Nevertheless, dissolved CO_2_ or carbonate was significantly produced in phases I and II, reaching up to 172 mg L^−1^, and then gradually decreased **(**Fig. 2c**)**. In contrast, DOC content progressively increased from 1262 to 3903 mg L^−1^ **(**Fig. 2c**)**, whereas pH decreased from 7.12 to 6.31, indicating the conversion of CO into liquid organics **(**Fig. 2e**)**, which was probably the cause of the pH drop. Furthermore, DN content sharply decreased in phase I from 528 to 355 mg L^−1^, implying active cell proliferation, and then slowly increased to 580 mg L^−1^ **(**Fig. 2d**)**. The concentration profiles of the produced carboxylates (acetate, propionate, *i*-butyrate, *n*-butyrate, *i*-valerate, *n*-valerate, *n*-caproate, *n*-heptylate, and *n*-caprylate) during the operation period are shown in Fig. 3.

### Production of MCCAs (up to C8) from gaseous CO by mixed culture

Although previous studies have attempted to integrate syngas fermentation and chain elongation, the present study is the first to achieve *n*-caprylate production directly from gaseous CO by mixed culture. The maximal *n*-caprylate concentration was 1.033 mmol L^−1^, with the highest production rate of 0.112 mmol L^−1^ day^−1^ (Table 1). In addition, maximal *n*-caproate concentration of 1.892 mmol L^−1^ was also noted, with the highest production rate of 0.276 mmol L^−1^ day^−1^. The other even-carbon-number carboxylates produced included acetate (80.649 mmol L^−1^) and *n*-butyrate (8.121 mmol L^−1^). Nonetheless, it must be noted that the MCCAs production rates and maximal concentrations obtained in the present study are not higher than those reported in previous works using pure cultures and/or syngas fermentation effluent (Table 2), which may be owing to insufficient substrate and toxicity of the carboxylate products. First, acetate is essential for MCCAs production because it is the initial feedstock of reversed β-oxidation, which is considered as the model reaction of chain elongation [20, 21]. Furthermore, Diender et al. [9] observed that addition of acetate significantly stimulated MCCAs production from CO. In contrast, in the present study, low-carbon-density gaseous CO was used as the solo substrate, which resulted in relatively low concentration of acetate in the liquid broth. Nevertheless, the MCCA concentrations and production rates obtained in the present study are comparable with those reported by Zhang et al. [15], who achieved conversion of gaseous CO_2_ and H_2_ to MCCAs without addition of acetate. Second, product toxicity has been generally recognized by researchers, and various extraction methods such as membrane extraction and electrolytic extraction have been developed [22–24]. In the present study, obvious product inhibition was observed during phase VII of operation, when *n*-caproate and *n*-caprylate production was almost stopped and the product concentrations remained unaltered (Fig. 3a).

**Table 1:** Average and maximal consumption/production rates of CO and main carboxylate products for the different operation phases

	Phase	CO	Even-carbon number carboxylates				Odd-carbon number carboxylates		
			Acetate	*N*-butyrate	*N*-caproate	*N*-caprylate	Propionate	*N*-valerate	*N*-heptylate
Average consumption/production rates (mmol L^−1^ day^−1^)	I	1.406 (0.671)^a^	0.146 (0.042)	0.004 (0.001)	0.000	0.000	0.056 (0.007)	0.000	0.000
	II	3.720 (0.724)	0.515 (0.072)	0.062 (0.005)	0.000	0.000	0.006 (0.009)	0.000	0.000
	III	5.033 (0.761)	0.532 (0.072)	0.092 (0.012)	0.000	0.000	0.033 (0.009)	0.000	0.000
	IV	6.774 (0.644)	0.272 (0.230)	0.074 (0.020)	0.000	0.000	− 0.003 (0.022)	0.060 (0.014)	0.000
	V	8.750 (0.519)	0.885 (0.264)	0.065 (0.004)	0.000	0.000	0.087 (0.020)	0.003 (0.013)	0.000
	VI	10.376 (0.793)	0.618 (0.320)	0.056 (0.030)	0.045 (0.006)	0.025 (0.003)	0.035 (0.015)	0.011 (0.024)	0.045 (0.006)
	VII	5.446 (3.666)	0.399 (0.143)	0.091 (0.012)	0.009 (0.002)	0.004 (0.001)	0.005 (0.005)	0.001 (0.001)	0.000 (0.002)
Maximal consumption/production rates (mmol L^−1^ day^−1^)		13.950	10.985	1.188	0.276	0.112	0.617	0.834	0.442
Maximal concentrations (mmol L^−1^)		/	80.649	8.121	1.892	1.033	4.424	1.669	1.635

^a^Standard deviations in brackets

**Table 2:** Maximal production rates compared with other similar works

Culture	Substrate	Reactor	Product extraction	Maximal production rates (mmol L^−1^ day^−1^)				Maximal concentrations (mmol L^−1^)				References
				C2^a^	C4	C6	C8	C2	C4	C6	C8	
*Clostridium autoethanogenum* + *Clostridium kluyveri*	Acetate + CO	Batch	No	NA^b^	8.5	2.5	ND	NA	~15.5	~1.6	ND	[9]
*Clostridium ljungdahlii* + *Clostridium kluyveri*	Acetate + syngas (60% CO, 35% H_2_, 5% CO_2_)	Continuous	Yes	NA	32.3	11.7	ND	NA	49.1	17.4	< 0.04	[10]
*Clostridium kluyveri*	Acetate + ethanol (actual syngas fermentation effluent)	Continuous	Yes	NA	9.4	39.9	1.4	NA	20.0	~56.0	2.2	[8]
Mixed culture	Acetate + ethanol (actual syngas fermentation effluent)	Anaerobic filter (semi-continuous)	No	NA	196.1	14.7	ND	NA	190.2	8.6	ND	[7]
Mixed culture	CO_2_ + H_2_	Hollow-fiber membrane biofilm reactor	No	3.25	0.65	0.27	0.12	123.3	21.4	8.4	2.9	[15]
Mixed culture	CO	Semi-continuous	No	11.0	1.2	0.3	0.10	80.6	8.1	1.9	1.0	This study

^a^C2, C4, C6, C8 are for acetate, *n*-butyrate, *n*-caproate and *n*-caprylate, respectively

^b^Negative rates are indicated NA not detected are indicated ND, “~” means the approximate value

It must be noted that the production of *n*-caproate and *n*-caprylate occurred at the end of phase VI of operation, with the highest concentrations obtained at the beginning of phase VII, indicating a lag phase of 96 and 100 days, respectively. Similarly, Richter et al. [10] reported a lag phase of 700 h for *n*-caproate production in a total operation period of 2200 h, while Zhang et al. [15] observed a lag phase of 37 and 65 days for *n*-caproate and *n*-caprylate production, respectively, in a total operation period of 80 days. These findings indicate that lag phases are quite common and that the elongation sequentially progresses to carboxylates of higher carbon number.

### Production of odd-carbon-number carboxylates

Chain elongation is achieved via the cyclic process of reversed β-oxidation, during which the carboxylates are elongated by two carbon atoms in every cycle [2, 25]. The reversed pathway could accept both even- and odd-chain substrates for carbon chain initiation and elongation [2, 25]. As most of the studies on chain elongation have employed acetate as the substrate and ethanol as the electron donor, only even-carbon-number carboxylates have been mainly discussed [24, 26–28]. Nevertheless, odd-carbon-number carboxylates, such as propionate, *n*-valerate, and *n*-heptanoate, have also been detected in these systems in small quantities with low proportions of production (propionate: 0–17.6 mmol L^−1^, 0–2%; *n*-valerate: 0–8.8 mmol L^−1^, 0–5%; *n*-heptanoate: 0–3.8 mmol L^−1^, 0–7%) [24, 26–28]. Liu et al. documented that hydrolysis of protein-rich biowaste produced propionate, which could be further elongated [17]. Furthermore, Grootscholten et al. [26]. successfully produced *n*-valerate and *n*-heptanoate from propionate by using ethanol as the electron donor. In the present study, propionate was synthesized continuously during the operation period (Fig. 3b), and the maximal propionate concentration obtained was 4.424 mmol L^−1^, with the highest production rate of 0.617 mmol L^−1^ day^−1^ (Table 1). While inoculum hydrolysis might contribute to a part of propionate production [17], the rest of the propionate produced might have been directly derived from CO fermentation, because comparable propionate production from syngas fermentation has been reported in a previous study by Liu et al. [29].

Furthermore, *n*-valerate (1.669 mmol L^−1^) and *n*-heptylate (1.635 mmol L^−1^) were also produced almost synchronously with *n*-caproate and *n*-caprylate during phase VII of operation, suggesting that lag phase also existed for *n*-valerate and *n*-heptanoate production. While a negligible increase in the concentration of *i*-butyrate from 1.573 to 2.042 mmol L^−1^ was observed during phase I–IV, the concentration remained almost unchanged, suggesting that *i*-butyrate was probably not the product of chain elongation (Fig. 3c). Furthermore, *i*-valerate was absent during phase I, but suddenly increased to 0.926 mmol L^−1^ at the beginning of phase II, and slowly reached 1.919 mmol L^−1^ on day 199 (Fig. 3c).

### Increase in CO partial pressure promoted CO utilization and product selectivity

The reactor performance varied during the seven phases of operation owing to different CO partial pressures. The average rates of CO consumption increased from phase I to VI with the increase in CO partial pressure (Table 2). Phases I–VII could be regarded as the period of gradual acclimation of the mixed culture to utilize CO as the substrate, and the toxicity of CO to the microorganisms could be alleviated by the presence of carboxydotrophic organisms [9]. However, the average CO utilization rate of phase VII decreased despite the supplementation of the same concentration of CO as that in phase VI (Table 1), which could possibly be owing to product inhibition.

Theoretically, production of methane was not expected because 2-BES was added to totally inhibit methanogenesis [18]. However, methane production was noted in phase VI and its concentration rapidly increased (Fig. 2a), suggesting that the inhibitor lost its efficacy to some extent during long-term operation. It has been reported that 2-BES could be consumed as a competing electron acceptor by sulfate-reducing bacteria or dehalogenating microorganisms in mixed culture [30, 31]. In the present study, hydrogen synthesis started from phase II and continued during phases III, IV, and V (Fig. 2a), whereas CO_2_ was produced in small quantity during phases I, II, and III and sharply increased during phases IV and V (Fig. 2a). Some of the CO_2_ produced might have contributed to the increase in DIC content during phases I and II as dissolved CO_2_ or carbonate/bicarbonate. In phase III, the DIC content decreased with the decline in pH (Fig. 2d, e). During phase VI, both H_2_ and CO_2_ produced were re-consumed by the mixed culture, which could also be used as substrate for MCCAs production [15].

**Fig. 4:**
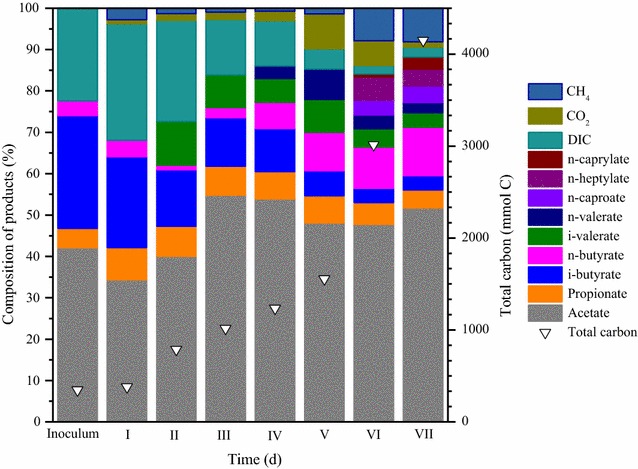
Composition of the products at the beginning and the end of seven incubation phases. The samples from day 0, 10, 20, 36, 44, 60, 100, 199 represent the inoculum, phase I, II, III, IV, V, VI and VII, respectively. Hollow triangle means the total carbons. Calculated in mmol C basis

Figure 4 shows the compositions of the produced carboxylates as well as the DIC, CO_2_, and CH_4_ contents in the initial culture and at the end of each operation phases, calculated based on mmol C. To estimate the carbon source provided by the dead cell decompose and yeast extract, cell elemental composition of C_5_H_7_NO_2_ and the data of DN (Fig. 2d) were used. The DN increases from 528 to 580 mg L^−1^, corresponding to a carbon increment of 18.6 mmol L^−1^. This is negligible (2.1%) compared with carbon increment of 890.3 mmol L^−1^ of CO consumption, Therefore, the following calculation ignored the carbon contribution from inoculum. While DIC, acetate, propionate, *i*-butyrate, and *n*-butyrate were detected in the initial culture, acetate was the dominant product, reaching 42.1% (day 0). The increase in CO partial pressure obviously broadened the products spectrum and also improved product selectivity. The selectivity of undesired products, such as *i*-butyrate, declined from 27.2% (day 0) to 3.5% (day 199). Furthermore, the proportion of DIC decreased from 22.4% (day 0) to 2.3% (day 199), whereas that of acetate remained at around 50%, with increasing absolute concentration (Fig. 3a). A similar phenomenon was also observed for propionate, with generally unchanged selectivity but increased absolute concentration (Fig. 3b). The selectivity of *n*-butyrate increased from 3.6% (day 0) to 11.7% (day 199), along with an increase in its absolute concentration. It must be noted that *n*-caproate, *n*-heptylate, and *n*-caprylate were first synthesized during phase VI, and exhibited selectivity of 3.9, 4.1, and 2.9%, respectively.

### Dominant microorganisms in the mixed culture

**Fig. 5:**
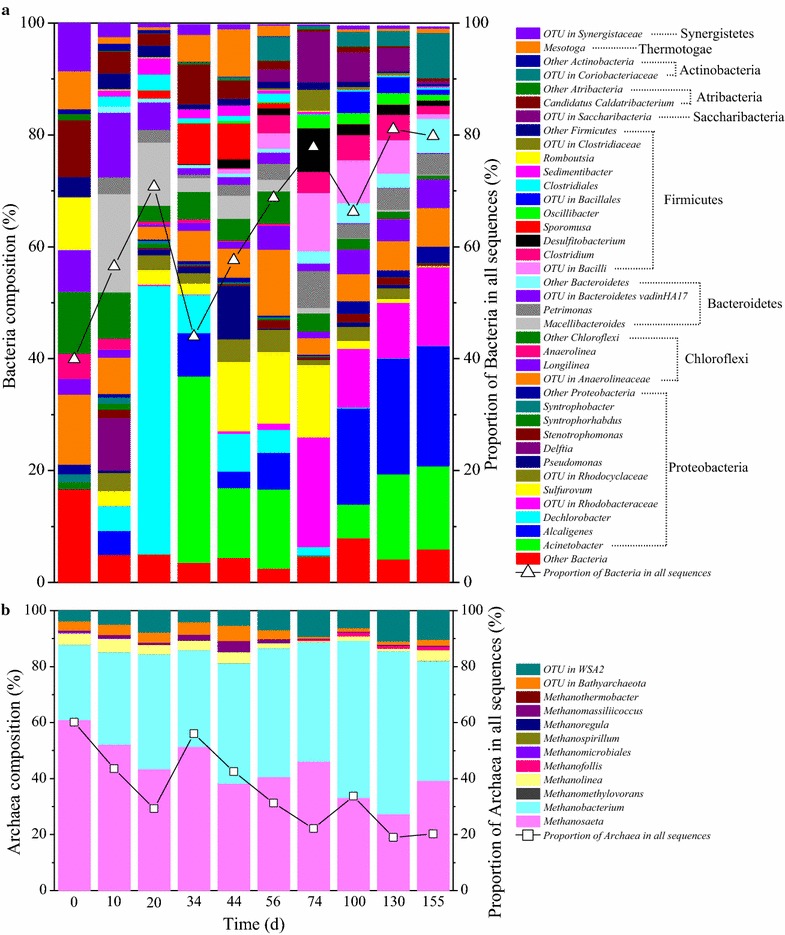
Taxonomic distribution of all the microbiomes at genus level. **a** Bacteria, **b** archaea. The samples from day 0, 10, 20, 34, 44, 56, 74 represent the inoculum, phase I, II, III, IV, V and VI, respectively, the samples from day 100, 130 and 155 represent phase VII

A total of 366,264 sequences with an average length of 273 bp were obtained from the 10 samples. The sequences were characterized and used for taxonomic classification against Silva database (Release128, http://www.arb-silva.de), and the taxonomic distribution of all the microbiomes at genus level is shown in Fig. 5. The genera with average relative abundance of more than 1% are listed in Additional file 1: Table S2. Overall, *Methanosaeta* (16.2% ± 9.4%), *Methanobacterium* (14.0 ± 3.7%), *Acinetobacter* (5.9 ± 5.5%), *Alcaligenes* (5.7 ± 6.4%), *Dechlorobacter* (4.5 ± 9.1%), and OTU (operational taxonomic units) from Rhodobacteraceae (4.3 ± 5.4%) were dominant at the genus level among the 10 samples.

**Fig. 6:**
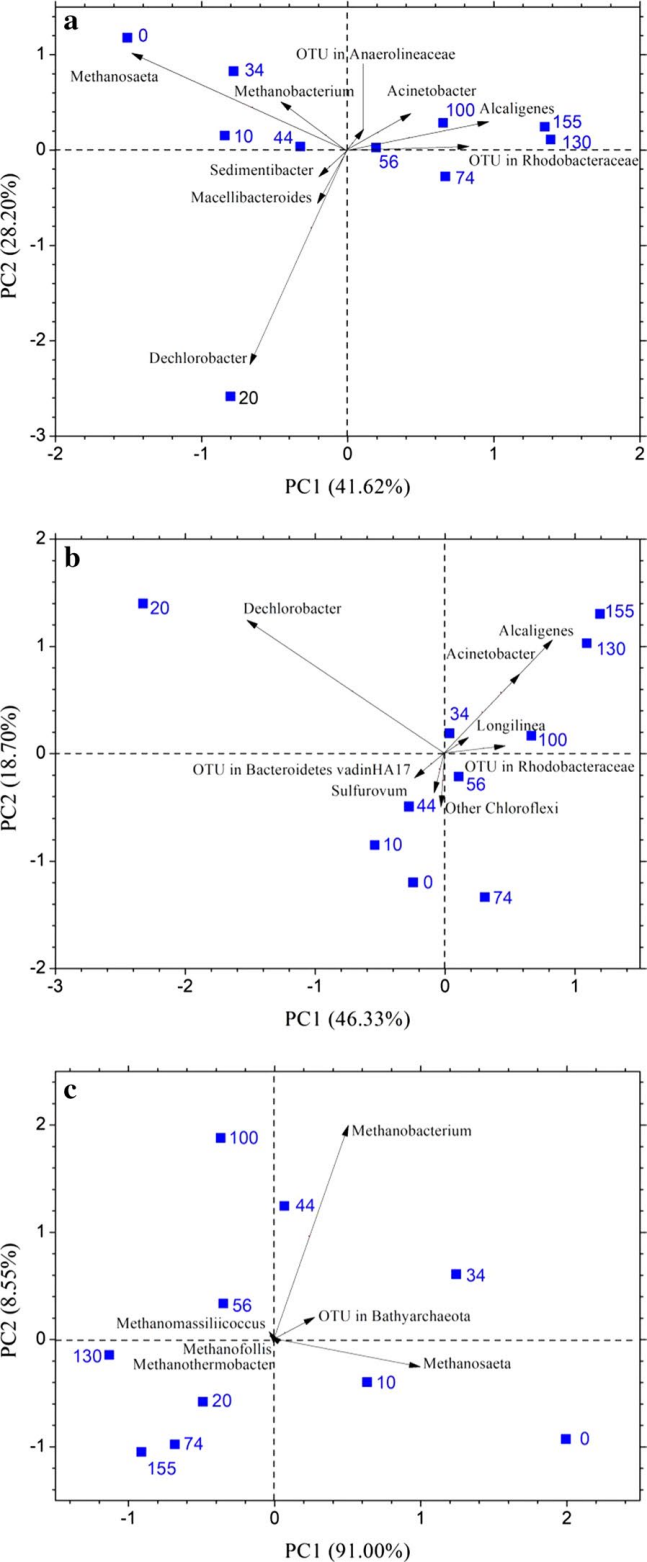
Principal component analysis based on the proportion of sequence numbers. **a** Total microbiomes. **b** Bacteria. **c** Archaea. The samples from day 0, 10, 20, 34, 44, 56, 74 represent the inoculum, phase I, II, III, IV, V and VI, respectively, the samples from day 100, 130 and 155 represent phase VII

To determine the response of the microbial structure to gradient partial pressures of CO, the 10 samples were subjected to principle component analysis (PCA) based on Bray–Curtis distance for total microbiomes, bacteria, and archaea (Fig. 6). The first two principal coordinates showed 69.8, 65.0, and 99.6% of the total variation, respectively. Based on the distribution distance, the results indicated that microbial diversity and community structure were closely related to CO concentration, especially with regard to the structure of bacteria. The close distance between samples collected on day 130 and 155 indicated establishment of a stable microbial community during the long-term operation period with CO as the sole substrate. Furthermore, sample collected on day 20 was distant from other samples, indicating distinctions in microbial community structures, which resulted from the outburst of *Dechlorobacter* in sample collected on day 20. *Dechlorobacter*, which is closest to the genus *Azospira*, is a (per)chlorate-reducing β-proteobacterium [32]. Thus, in the present study, a novel strain isolated from paper mill waste might have possibly existed in the mixed culture and might belong to the genus *Dechlorobacter,* which can use acetate, propionate, and butyrate as electron donors and chlorate as electron acceptor [33]. These chlorate-reducing microorganisms in the mixed culture could use 2-BES as electron acceptor because the tetrahedral structure of 2-BES with three oxygen bonds is similar to chlorate. In a previous study by Steinbusch et al. [28], *Dechlorosoma oryzae*, which could also use 2-BES as methanogenesis inhibitor, was found to be dominant in the chain-elongating mixed culture.

As expected, the relative abundance of the major methanogens *Methanosaeta* and *Methanobacterium* decreased from 36.6 to 8.0% and from 16.1 to 8.6% in all the microbiomes with increasing acclimation time, respectively, because methanogenesis was inhibited by 2-BES. Furthermore, the proportion of archaea in the microbiomes declined from 60.1 to 20.2%, while their composition remained almost unchanged (Fig. 5b). Thus, archaea in the mixed culture had an insignificant relationship with CO consumption or MCCAs production, which was further confirmed by the relative decentralized distribution of the samples in PCA of archaea (Fig. 6c).

### Microorganisms responsible for MCCAs production from CO

**Fig. 7:**
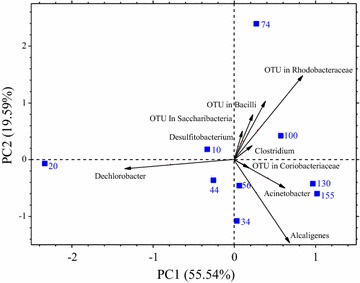
Principal component analysis based on the ratio of relative abundance of microbes compared with the inoculum culture. The samples from day 10, 20, 34, 44, 56, 74 represent phase I, II, III, IV, V and VI, respectively, the samples from day 100, 130 and 155 represent phase VII

The microorganisms exhibiting an increase in abundance along with the incubation period could be effectively responsible for MCCAs production from CO. To exclude background interference from the initial inoculum, PCA based on the ratio of relative abundance of microorganisms was performed and the results were compared with those of the initial inoculum culture (Fig. 7). *Alcaligenes*, *Acinetobacter*, OTU from Rhodobacteraceae*, Clostridium*, *Desulfitobacterium,* and OTU from Bacilli, Saccharibacteria, and Coriobacteriaceae were identified to be responsible for MCCAs production from CO because they were closely abundant in the samples from phases VI and VII.

Interestingly, the relative abundance of *Acinetobacter*, *Alcaligenes,* and OTU from Rhodobacteraceae significantly increased (Figs. 5a, 6a, b, 7), which was unexpected based on previous reports on syngas fermentation and chain elongation. *Acinetobacter calcoaceticus*, the dominate species of the genus *Acinetobacter*, which accounted for more than 49.1% of the sequences from *Acinetobacter* in the present study, is known to produce fatty alcohols and acids. This bacterium possesses the gene encoding fatty acyl-CoA reductases, which play significant role in reversed β-oxidation pathway for MCCAs production [34, 35]. Furthermore, the wax ester synthase/acyl-CoA: diacylglycerol acyltransferase of *Acinetobacter baylyi* catalyzes the esterification of ethanol with acyl-CoA [34, 35]. It has been reported that *Acinetobacter* strains could grow on CO, and produce carbon monoxide dehydrogenase (CODH), the key enzyme that catalyzes CO conversion [36]. Therefore, *Acinetobacter* could be one of the microorganisms that could simultaneously utilize CO and participate in chain elongation. Kucek et al. [6] also found OTUs for *Acinetobacter* spp. that were predominant and increased in dominance up to 55.5% of the relative abundance of their chain elongation works.

Among *Alcaligenes* spp., including *A. aquatilis*, *A. faecalis,* and *A. eutrophus*, *A. eutrophus* is well known for its ability to synthesize poly-β-hydroxybutyrate. Furthermore, *A. eutrophus* could synthesize polyhydroxyalkanoates under the conditions of growth inhibition by CO [37], and the Ni–Fe hydrogenase of *A. eutrophus* is tolerant to CO [38]. Moreover, the CODH gene has also been observed in the genome of *A. faecalis* and *A. eutrophus* (http://www.uniprot.org). It has been reported that the genes that are crucial for MCCAs production, such as β-ketothiolase and NADPH-linked acetoacetyl-CoA reductases, are abundant and actively expressed in *A. eutrophus* [39–41], and that genes encoding long-chain acyl-CoA synthetase are abundant in *Alcaligenes* genomes (http://www.uniprot.org).

Rhodobacteraceae is mainly found in marine environment, and nearly all of its members harbor the CODH gene, indicating their ability to oxidize CO [39–41]. Thus, the OTU from Rhodobacteraceae detected in the present study may also contribute to CO utilization. A previous study reported that Rhodobacteraceae possesses genes required for the biosynthesis of indole 3-acetic acid [42], and that members of this family could produce secondary metabolites such as phenylacetic acid and tropodithietic acid [43]. However, these microorganisms have been rarely reported in chain elongation studies, and the enzymes required for reversed β-oxidation, such as fatty acyl-CoA synthase and long-chain acyl-CoA synthetase, have all been discovered in the genomes of Rhodobacteraceae (http://www.uniprot.org).

The sample collected on day 100 (phase VII) was important because *n*-valerate, *n*-caproate, *n*-heptylate, and *n*-caprylate were all produced at a high rate around this period (Fig. 3a, b). *Clostridium* spp. were observed in small quantity (< 0.2%) during phases I–IV, and increased up to 3.6% on day 100 (see Additional file 1: Table S2). It is known that *Clostridium* spp. are associated with carboxylates metabolism, and *C. kluyveri* has been the most studied and considered as the model microorganism for chain elongation by reversed β-oxidation [20, 21]. However, the relative abundance of *Clostridium* spp. in the present study was quite low, when compared with those reported by Zhang et al. [15] (47.6% of *C. ljungdahlii*, *C. autoethanogenum,* and *C. kluyveri*), Algar et al. [1] (50% of *C. ljungdahlii* and *C. kluyveri*), and Steinbusch et al. [28] (57.8% of *C. kluyveri*). Nonetheless, although the abundance of *Clostridium* spp. was not as high as expected, the increase in the relative abundance of *Clostridium* spp. was positively correlated with MCCAs production. Some members of *Clostridium* spp., such as *C. ljungdahlii* and *C. autoethanogenum,* are known to be syngas utilizer. *C. autoethanogenum* is one of the model organisms for syngas metabolism and can convert CO or syngas to ethanol and acetate [44], while *C. ljungdahlii* is also known as a typical carboxydotrophic bacterium [45]. In the present study, the sequences from *Clostridium* spp. were assigned to *C. kluyveri*, *C. ljungdahlii,* and *C. autoethanogenum*, which might have played a significant role in CO utilization as well as chain elongation in the mixed culture. Based on the results of microbial analysis, it can be concluded that *Acinetobacter*, *Alcaligenes,* and OTU from Rhodobacteraceae could harbor the potential for both CO utilization and chain elongation, and could achieve MCCAs production from CO as the sole substrate, besides *Clostridium* spp. that are well known for their ability of CO utilization and chain elongation.

## Conclusions

In the present study, the feasibility of one-step C6–C8 MCCAs production from CO as the sole substrate without additional electron donors was confirmed. Although fermentation of CO into C2–C4 SCCAs was not difficult and could be directly initiated with an anaerobic biogas-producing microbiome, the process required long acclimation period of up to 100 days to obtain a microbiome capable of directly converting CO to C6–C8 MCCAs. In the first five phases of operation, acetate and butyrate production reached 50 and 6 mmol L^−1^, respectively, and no *n*-caproate, *n*-heptylate, or *n*-caprylate was detected. It must be noted that a similar inoculum was used in our previous study to initiate chain elongation of acetate and ethanol in not more than 20 days [16]. Therefore, it can be concluded that the shortage of CO-utilizing chain-elongating microorganisms, and not the lack of SCCAs-utilizing chain-elongating microorganisms or inadequate SCCAs as substrate, resulted in the absence of C6–C8 acids. In the first 90 days, 64% of the produced acids were acetate and the rest were C3–C5 products. In contrast, during 90–199 days, *n*-caproate, *n*-heptylate, and *n*-caprylate contributed to 13, 14, and 10% of the newly produced acids, respectively, when compared with acetate (45%) and *n*-butyrate (13%), confirming that phase VII operation was established with CO-tolerant and CO-utilizing chain-elongating microbiomes.

Overall, the present study is a proof-of-principle that the carboxylate and syngas platform could be integrated in a shared growth vessel, and could become a promising method to convert syngas to preferable liquid biochemicals, which is significant as a new starting point for value-added exploitation of syngas. Although further studies are needed before its practical application, including improvement of production yield and selectivity, improvement of gas–liquid mass transfer, control of products formation, onsite products separation and purification technologies, etc., the concept of one-step MCCAs production from CO could evoke a new window for syngas biorefinery and necessitate coordinating the cooperation of syngas fermenters and chain elongators. Moreover, one-step MCCAs production from CO can be realized in a single cell, and the biorefinery potentials of the speculated CO-utilizing chain-elongating microorganisms, such as *Alcaligenes*, *Acinetobacter*, and OTU from Rhodobacteraceae, in addition to *Clostridium* spp., need to be further exploited.

## Additional file

**Additional file 1: Table S1.** Quality and sequence number of the extracted DNA. **Table S2.** Taxonomic distribution of the 10 samples at genus level (only relative abundance > 1%).
