# Supplementary material for: One-step production of C6–C8 carboxylates by mixed culture solely grown on CO
Source: Biotechnol Biofuels. 2018 Jan 9;11:4. doi: 10.1186/s13068-017-1005-8 (PMC5761104; doi:10.1186/s13068-017-1005-8)
Supplement: Supplementary file 1 — Additional file 1: Table S1. Quality and sequence number of the extracted DNA. Table S2. Taxonomic distribution of the 10 samples at genus level (only relative abundance > 1%). [file 13068_2017_1005_MOESM1_ESM.docx]

**Additional file for:**

**One-step Production of C6–C8 Carboxylates by Mixed Culture Solely Grown on CO**

*Pinjing He^a,b,c^, Wenhao Han^a^, Liming Shao^b,c^, Fan Lü^a,b*^*

*^a^* State Key Laboratory of Pollution Control and Resource Reuse, Tongji University, Shanghai 200092, China

*^b^* Institute of Waste Treatment and Reclamation, Tongji University, Shanghai 200092, China

*^c^* Centre for the Technology Research and Training on Household Waste in Small Towns & Rural Area, Ministry of Housing and Urban-Rural Development of PR China (MOHURD), China

*** Corresponding author: [lvfan.rhodea@tongji.edu.cn](mailto:lvfan.rhodea@tongji.edu.cn) (Fan Lü)

**Table S1.** Quality and sequence number of the extracted DNA.

| Sample | DNA concentration (ng/μl) | OD260/280 | OD260/230 | Sequence number |
| --- | --- | --- | --- | --- |
| Day 0 | 78.0 | 1.59 | 0.64 | 37075 |
| Day 10 | 99.7 | 1.74 | 1.05 | 34405 |
| Day 20 | 48.8 | 1.70 | 0.80 | 38271 |
| Day 34 | 65.2 | 1.66 | 0.80 | 34533 |
| Day 44 | 42.9 | 1.66 | 0.80 | 34743 |
| Day 56 | 55 | 1.71 | 0.85 | 30780 |
| Day 74 | 45.5 | 1.69 | 0.86 | 44934 |
| Day 100 | 60.9 | 1.65 | 1.11 | 36557 |
| Day 130 | 47.9 | 1.81 | 1.13 | 41216 |
| Day 155 | 59.4 | 1.81 | 1.35 | 33750 |

**Table S2.** Taxonomic distribution of the 10 samples at genus level (only relative abundance >1%).

| Genus | Day 0  (%) | Day 10  (%) | Day 20  (%) | Day 34  (%) | Day 44  (%) | Day 56  (%) | Day 74  (%) | Day 100  (%) | Day 130  (%) | Day 155  (%) | Average  (%) |
| --- | --- | --- | --- | --- | --- | --- | --- | --- | --- | --- | --- |
| *Methanosaeta* | 36.6 | 22.7 | 11.8 | 28.0 | 15.9 | 12.5 | 10.3 | 11.2 | 5.2 | 8.0 | 16.2 ± 9.4 |
| *Methanobacterium* | 16.1 | 14.3 | 11.1 | 18.8 | 17.8 | 14.2 | 9.5 | 19.0 | 10.9 | 8.6 | 14.0 ± 3.7 |
| *Acinetobacter* | 0.0 | 0.0 | 0.0 | 14.3 | 7.0 | 9.6 | 0.1 | 4.0 | 12.2 | 11.9 | 5.9 ± 5.5 |
| *Alcaligenes* | 0.0 | 2.4 | 0.0 | 3.3 | 1.6 | 4.5 | 0.1 | 11.4 | 16.5 | 17.2 | 5.7 ± 6.4 |
| *Dechlorobacter* | 0.0 | 2.5 | 31.4 | 2.9 | 3.8 | 2.8 | 1.1 | 0.1 | 0.1 | 0.0 | 4.5 ± 9.1 |
| OTU in Rhodobacteraceae | 0.0 | 0.0 | 0.1 | 0.1 | 0.3 | 0.8 | 15.4 | 7.0 | 7.9 | 11.3 | 4.3 ± 5.4 |
| OTU from family Anaerolineaceae | 4.4 | 3.5 | 1.4 | 2.1 | 2.8 | 7.6 | 2.2 | 3.1 | 4.0 | 5.5 | 3.7 ± 1.7 |
| *Sulfurovum* | 0.0 | 1.5 | 1.7 | 0.8 | 7.0 | 8.7 | 10.1 | 0.9 | 0.5 | 0.1 | 3.1 ± 3.7 |
| *Macellibacteroides* | 0.0 | 9.9 | 7.4 | 1.1 | 2.3 | 1.4 | 0.8 | 0.1 | 0.3 | 0.1 | 2.3 ± 3.3 |
| OTU from class Bacilli | 0.0 | 0.0 | 0.0 | 0.0 | 0.5 | 1.9 | 8.1 | 5.1 | 4.8 | 0.7 | 2.1 ± 2.7 |
| *Petrimonas* | 0.0 | 1.7 | 1.5 | 0.3 | 1.1 | 2.0 | 5.2 | 1.7 | 3.1 | 3.0 | 2.0 ± 1.4 |
| *Longilinea* | 1.1 | 0.8 | 0.3 | 0.6 | 0.7 | 2.9 | 0.9 | 2.9 | 3.1 | 4.0 | 1.7 ± 1.3 |
| OTU from phylum Chloroflexi | 2.0 | 3.2 | 1.4 | 1.6 | 1.5 | 3.2 | 2.2 | 1.1 | 0.9 | 0.4 | 1.7 ± 0.9 |
| OTU from class Bacteroidetes | 3.0 | 6.5 | 3.2 | 0.5 | 0.8 | 1.4 | 1.1 | 0.1 | 0.1 | 0.1 | 1.7 ± 1.9 |
| OTU from phylum Saccharibacteria | 0.0 | 0.0 | 0.0 | 0.0 | 0.0 | 1.5 | 7.1 | 3.5 | 3.3 | 0.4 | 1.6 ± 2.2 |
| Candidatus *Caldatribacterium* | 4.1 | 2.2 | 1.4 | 3.0 | 1.8 | 1.0 | 0.3 | 0.7 | 0.3 | 0.4 | 1.5 ± 1.2 |
| OTU from family *Coriobacteriace* | 0.0 | 0.0 | 0.0 | 0.0 | 0.2 | 2.9 | 0.4 | 1.7 | 2.1 | 6.4 | 1.4 ± 2.0 |
| *Clostridium* | 0.2 | 0.0 | 0.0 | 0.0 | 0.1 | 2.2 | 3.0 | 3.0 | 3.6 | 1.2 | 1.3 ± 1.4 |
| *Mesotoga* | 2.7 | 0.7 | 0.3 | 2.0 | 4.8 | 1.3 | 0.0 | 0.3 | 0.3 | 0.7 | 1.3 ± 1.4 |
| OTU from phylum Bathyarchaeota | 1.9 | 1.6 | 1.0 | 2.5 | 2.3 | 1.0 | 0.1 | 0.4 | 0.2 | 0.4 | 1.1 ± 0.8 |
| *Desulfitobacterium* | 0.0 | 0.0 | 0.0 | 0.0 | 0.9 | 0.8 | 6.1 | 1.3 | 1.5 | 0.8 | 1.1 ± 1.7 |
| *Methanolinea* | 2.5 | 2.1 | 0.9 | 1.8 | 1.6 | 0.5 | 0.1 | 0.5 | 0.2 | 0.8 | 1.1 ± 0.8 |
| *Thauera* | 0.0 | 1.7 | 1.5 | 0.6 | 1.6 | 1.9 | 0.2 | 1.6 | 1.5 | 0.0 | 1.1 ± 0.7 |
| *Thermincola* | 0.0 | 0.0 | 7.6 | 1.7 | 0.9 | 0.1 | 0.0 | 0.0 | 0.0 | 0.0 | 1.0 ± 2.3 |
